# Coronaviruses: Molecular and Cellular Biology

**DOI:** 10.3201/eid1404.080016

**Published:** 2008-04

**Authors:** Julian L. Leibowitz

**Affiliations:** * Texas A&M College of Medicine, College Station, Texas, USA

**Keywords:** Coronaviruses, molecular biology, book review

## Abstract

Coronaviruses: Molecular and Cellular Biology

Coronaviruses are a group of single-stranded RNA viruses that mainly cause enteric and respiratory diseases in infected hosts. Before 2002, coronaviruses were known as important veterinary pathogens, as well as a cause of the common cold in humans. In 2002–2003, with the advent of the outbreak of severe acute respiratory syndrome (SARS), this picture changed. SARS was quickly shown to be caused by a novel coronavirus, and the ensuing explosion of research on coronaviruses is reflected in this new book.

This multi-authored book contains 16 chapters and is organized into 2 sections. The first section of 7 chapters covers most aspects of coronavirus replication, from virus binding and entry into the cell to genome packaging. When appropriate, these chapters also draw on recent work with the closely related arteriviruses. Each chapter generally offers excellent and balanced reviews of the coronavirus literature through 2006, with a few references from 2007. The second section of 9 chapters discusses various aspects of the host-pathogen interface of several coronaviruses; the major focus is the SARS coronavirus, although the human coronavirus NL63 and murine, feline, and avian coronaviruses are also covered.

This book provides a one-stop entry into current thinking in the field. For those unfamiliar with coronaviruses, the first section offers a current view of how these viruses replicate. Two areas that are not as well represented in this section are effects of coronavirus infection on cellular processes, such as the cell cycle, apoptosis, and other signaling pathways, and protein trafficking, virus assembly, and release. Separate chapters on these areas would have strengthened the book. In some ways, the second section of the book is not as satisfying. The 3 chapters on SARS coronavirus and the chapter on human coronavirus vaccine development have introductory sections that are somewhat repetitive. Including chapters on transmissible gastroenteritis virus and porcine respiratory coronavirus would also have been beneficial. That said, the chapters on SARS and the avian, murine, and feline coronaviruses are excellent. 

I heartily recommend that this book be placed in the library of every laboratory that is working on this fascinating group of viruses. It will be particularly valuable to newcomers to the field by providing a single entry point to recent thinking about these agents.

